# Age-related hearing loss accelerates cerebrospinal fluid tau levels and brain atrophy: a longitudinal study

**DOI:** 10.18632/aging.101971

**Published:** 2019-05-22

**Authors:** Wei Xu, Can Zhang, Jie-Qiong Li, Chen-Chen Tan, Xi-Peng Cao, Lan Tan, Jin-Tai Yu, Alzheimer’s Disease Neuroimaging Initiative

**Affiliations:** 1 Department of Neurology, Qingdao Municipal Hospital, College of Medicine and Pharmaceutics, Ocean University of China, Qingdao, China; 2 Genetics and Aging Research Unit, Mass General Institute for Neurodegenerative Diseases (MIND), Department of Neurology, Massachusetts General Hospital, Harvard Medical School, Boston, MA 02114, USA; 3 Department of Neurology, Qingdao Municipal Hospital, Qingdao University, Qingdao, China; 4 Clinical Research Center, Qingdao Municipal Hospital, Qingdao, China; 5 Department of Neurology and Institute of Neurology, Huashan Hospital, Shanghai Medical College, Fudan University, Shanghai, China; 6 Data used in preparation of this article were obtained from the Alzheimer’s Disease Neuroimaging Initiative (ADNI) database (adni.loni.usc.edu). As such, the investigators within the ADNI contributed to the design and implementation of ADNI and/or provided data but did not participate in analysis or writing of this report. A complete listing of ADNI investigators can be found at: http://adni.loni.usc.edu/wp-content/uploads/how_to_apply/ADNI_Acknowledgement_List.pdf

**Keywords:** age-related hearing loss, Alzheimer’s disease, biomarker, tau, MRI

## Abstract

Age-related hearing loss (ARHL) has been considered as a promising modifiable risk factor for cognitive impairment and dementia. Nonetheless, it is still unclear whether age-related hearing loss associates with neurodegenerative biomarkers of Alzheimer’s disease (AD). Participants with ARHL were selected from the established Alzheimer’s Disease Neuroimaging Initiative (ADNI) database. In multivariable models, the cross-sectional and longitudinal associations of ARHL with CSF β-amyloid (Aβ) and tau measurements, brain Aβ load, and cortical structural measures were explored. ARHL was associated with higher CSF levels of tau (p < 0.001) or ptau181 (p < 0.05) at baseline as well as faster elevation rates of these two types of biomarkers (p < 0.05). Although the baseline volume/thickness of hippocampus (p < 0.05) and entorhinal cortex (p < 0.0005) were higher in individuals with ARHL, these two regions (p < 0.01 for hippocampus, p < 0.05 for entorhinal cortex) displayed significantly accelerated atrophy in individuals with ARHL. No association of ARHL with CSF or brain Aβ levels was found. Subgroup analyses indicated that the above effects of ARHL were more significant in non-demented stage. Age-related hearing loss was associated with elevated cerebrospinal fluid tau levels and atrophy of entorhinal cortex.

## INTRODUCTION

Age-related hearing loss (ARHL) and dementia are two important global health concerns [1, 2]. ARHL has been considered as a promising modifiable risk factor for cognitive impairment [3–5]. Nonetheless, the relationship between ARHL and Alzheimer’s disease (AD) is still controversial [3]. Besides the insufficient statistical power due to small sample size, their relationship might be further complicated by misclassification bias due to misdiagnosis, given that (1) AD was defined in previous observational studies mostly without pathological evidence, such as amyloid PET imaging or cerebrospinal fluid (CSF) biomarkers; (2) aged subjects with hearing loss (HL) might be more intellectually capable than what the cognitive tests suggest [6]. Therefore, investigating the association between ARHL and AD biomarkers might be less biased and more informative about the causal relationship.

In recent AD criteria [7, 8], the levels of CSF Aβ_1-42_, tau, p-tau_181_, as well as PET imaging results have been established as core AD biomarkers to define the progressive stages in AD continuum, including preclinical AD, mild cognitive decline (MCI) due to AD, and dementia due to AD. Also, longitudinal observational studies suggested that the above biomarkers [9–12] and their ratios (tau/Aβ_1-42_ [13, 14] and p-tau_181_/Aβ_1-42_ [15, 16]) are good predictors of cognitive decline. In the AD continuum, the abnormal accumulation of these pathological proteins will finally lead to the structural atrophy and functional loss of specific brain regions, particularly the hippocampus and entorhinal cortex. In the present study, we aimed to explore how ARHL can influence these neurodegenerative biomarkers in the Alzheimer’s continuum based on the Alzheimer’s Disease Neuroimaging Initiative (ADNI) database.

## RESULTS

### ARHL and CSF biomarkers

**Figure 1:**
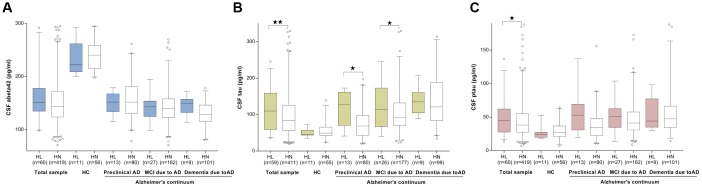
**The cross-sectional associations between ARHL and CSF biomarkers.** No association was found between ARHL and CSF Aβ_42_ levels (**A**). ARHL was associated with higher levels of CSF total tau (**B**) and ptau_181_ (**C**). All the above analyses were adjusted for age, gender, education, *APOE*4 status, pathological diagnosis of AD, DM2, hypertension, hyperlipidemia, BMI, and extracted CSF volume. *p value was calculated for the total sample.

We first investigated the association of ARHL with the baseline levels of the CSF Aβ and tau proteins in total group. CSF measurements of Aβ_42_, tau, and ptau_181_ were available for 479 participants (67 HC, 209 preclinical AD, 93 MCI due to AD, and 110 dementia due to AD), of whom 60 had ARHL (Table 1). No association was found between ARHL and CSF Aβ_42_ levels (β = 0.002, *p* = 0.28). (Figure 1A) ARHL was associated with higher levels of CSF total tau (β = 0.23, *p* = 0.002) and ptau_181_ (β = 0.17, *p* = 0.017) after adjustment for age, gender, education, *APOE*4 status, pathological diagnosis, DM2, hypertension, hyperlipidemia, BMI, and extracted CSF volume. (Figure 1B–1C) CSF levels of tau (111 ± 54 pg/ml, 95% confidence interval (CI) = 95.6 to 124.6 pg/ml) and ptau_181_ (49 ± 26 pg/ml, 95% CI = 42 to 55 pg/ml) were significantly higher in individuals with ARHL than those in HN group (100 ± 61 pg/ml, 95% CI = 94 to 106 pg/ml for tau; 45 ± 26pg/ml, 95% CI = 42 to 47 pg/ml for ptau_181_). As for the biomarker ratios, ARHL was associated with higher CSF total tau/Aβ_42_ ratio (β = 7.96, *p* = 0.01), but not with ptau_181_ /Aβ_42_ ratio (β = 0.15, *p* = 0.07). (Supplementary Figure 1)

**Table 1:** Participant characteristics at baseline.

	**CSF Aβ42, tau, and ptau**			**Amyloid PET**			**Hippocampus**			**Other ROIs^#^**		
	**HL**	**HN**	***p* value**	**HL**	**HN**	***p* value**	**HL**	**HN**	***p* value**	**HL**	**HN**	***p* value**
No.	60	419		106	560		131	746		74	440	
Age (Mean ± SD, year)	77.4 ± 5.0	72.7 ± 7.1	<0.0001	77.3 ± 5.2	72.4 ± 6.9	<0.0001	77.5 ± 5.1	73.0 ± 6.9	< 0.0001	77.2 ± 5.6	72.5 ± 6.9	<0.0001
Gender (M/F)	50/15	220/199	0.0002	70/36	265/295	0.0004	90/41	362/384	< 0.0001	53/21	201/239	<0.0001
Education (Mean ± SD, year)	16.2 ± 3.0	15.9± 2.7	0.46	16.5 ± 2.8	16.3 ± 2.6	0.38	16.3± 2.9	16.0± 2.7	0.27	16.3 ± 2.9	15.9 ± 2.8	0.25
APOE Ɛ4 carrier status (0/1/2)	37/19/4	180/180/61	0.0174	65/33/8	261/230/68	0.02	79/42/10	355/301/90	0.02	41/27/6	198/188/54	0.22
Hypertension (yes/no)	26/34	194/225	0.67	49/57	257/303	0.95	66/65	343/403	0.35	39/35	188/252	0.11
Hyperlipidemia (yes/no)	29/31	217/202	0.62	60/46	286/274	0.30	68/63	379/367	0.82	34/40	208/232	0.83
BMI (Mean ± SD, kg/m^2^)	26.2 ± 4.1	26.8 ± 5.1	0.43	26.4 ± 4.4	27.4± 5.4	0.06	26.5 ± 4.6	27.0± 5.0	0.31	26.2 ± 4.2	26.6 ± 4.9	0.46
DM2 (yes/no)	4/56	29/390	0.94	10/96	54/506	0.95	15/126	63/683	0.40	7/67	36/404	0.71
CSF volume (ml)	19.0 ± 4.7	18.5 ± 5.1	0.44	…	…	…	…	…	…	…	…	…
Disease continuum												
Healthy control	11	56	0.13^&^	17	76	0.13^&^	30	164	0.15^&^	17	94	0.21&
Preclinical AD	27	182		33	147		27	123		15	70	
MCI due to AD	13	80		41	229		56	295		30	163	
Dementia due to AD	9	101		15	108		18	164		12	113	

Abbreviations: HL = Hearing Loss; HN = Hearing Normal; AD = Alzheimer’s Disease; BMI = Body Mass Index; DM2 = Diabetes Mellitus Type 2; ROI = Region of Interest

^#^Other five ROIs include middle temporal cortex, entorhinal cortex, parahippocampal area, posterior cingulate, and precuneus

^&^Chi-square test for trend of proportion of HL across disease continuum groups

When stratified by AD continuum group, as expected, CSF Aβ_42_ levels decreased and tau (total tau or ptau_181_) levels increased across groups from HC through preclinical AD and MCI to dementia. ARHL was associated with a higher level of CSF total tau only among those at preclinical and MCI stages (Figure 1A), but was not associated with CSF ptau_181_ or Aβ_42_ in any group (Figure 1B–1C). Potential interactions with *APOE*4 status and gender were found for CSF Aβ_42_ and total tau (p=0.07), respectively. Subgroup analyses showed that ARHL was associated with higher levels of CSF tau or ptau_181_ only among *APOE*4 carrier (β = 0.24, *p* = 0.03 for tau; β = 0.25, *p* = 0.03 for ptau_181_) and male (β = 0.30, *p* = 0.001 for tau; β = 0.19, *p* = 0.03 for ptau_181_) groups (Supplementary Figure 2).

**Figure 2:**
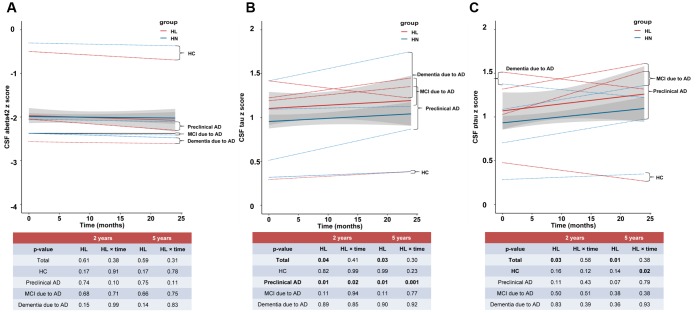
**The longitudinal influences of ARHL on CSF biomarkers.** ARHL at baseline was not associated with the change rate of Aβ_42_ (**A**), but with the faster elevation rates of CSF tau (**B**) and ptau_181_ (**C**). All the above analyses were adjusted for age, gender, education, *APOE*4 status, pathological diagnosis of AD, DM2, hypertension, hyperlipidemia, BMI, and extracted CSF volume. *p value was calculated for the total sample.

We next studied whether ARHL was associated with the changes of Aβ and tau levels through longitudinal analyses of the following 24 month widow from the baseline. ARHL was not associated with the change rate of Aβ_42_ (Figure 2A), but with the faster elevation rates of CSF tau (Figure 2B) and ptau_181_ (Figure 2C) in HC or preclinical AD, after adjustment for age, gender, education, *APOE*4 status, DM2, hypertension, hyperlipidemia, BMI, and extracted CSF volume. Similar results were concluded when the follow-up window was extended to 5 years.

### ARHL and amyloid PET

We then investigated the association of ARHL with the molecular imaging results. Florbetapir PET scans were available for 666 participants (93 HC, 180 preclinical AD, 270 MCI, and 123 AD), of whom 106 had ARHL (Table 1). As expected, a florbetapir cortical summary measurement (SUVR) increased across groups through the AD continuum. We did not identify a statistically significant association between ARHL and (summary or regional) SUVR when adjusting for all variables mentioned above. Similarly, no associations were detected between ARHL and SUVR when the analyses were stratified by AD continuum group. (Supplementary Figure 3)

### ARHL and MRI biomarkers

**Figure 3:**
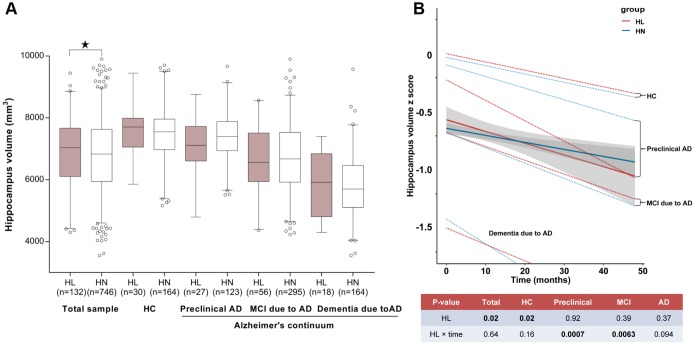
**The relationship between ARHL and hippocampus volume.** ARHL was associated with larger volume of hippocampus. Nonetheless, when stratified by AD continuum stage, no association was found in any group. (**A**) ARHL was associated with more rapid cortical thinning, especially in the preclinical or prodromal AD stage (**B**). All analyses were adjusted for age, gender, education, *APOE*4 status, pathological diagnosis of AD (for total sample), DM2, hypertension, hyperlipidemia, BMI, and ICV. *p value was calculated for the total sample.

**Figure 4:**
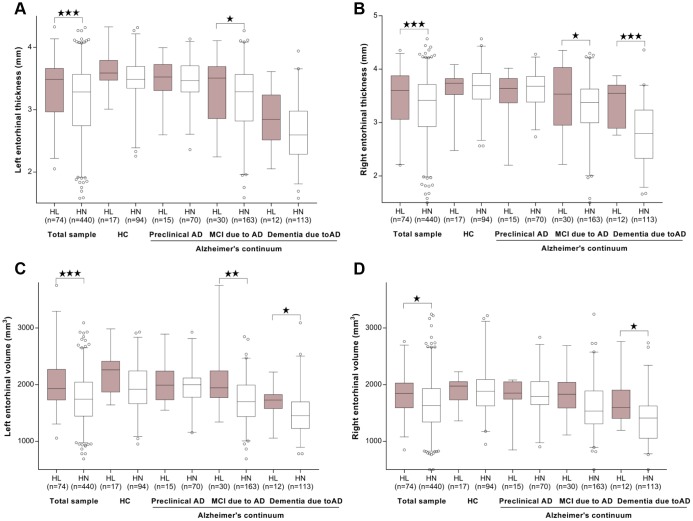
**The cross-sectional associations between ARHL and structural measurements of entorhinal cortex.** ARHL was associated with higher thickness/volume of entorhinal cortex. The significant associations with higher entorihnal cortex at baseline were observed only in MCI and/or dementia stage. (**A**–**D**) All analyses were adjusted for age, gender, education, *APOE*4 status, pathological diagnosis of AD, DM2, hypertension, hyperlipidemia, BMI, and intracranial volume. *p value was calculated for the total sample.

Cortical thickness or volume measures were available in 877 participants for hippocampus and 514 participants for another 20 ROIs. As expected, the cortical measures were greatest in HC, followed by those at the preclinical stage, and then those with MCI and dementia due to AD. In the fully-adjusted model, ARHL was associated with higher volume of hippocampus (β = 188.8, *p* = 0.04) (Figure 3A) and thickness/volume of parahippocampus (β = 8.99, *p* = 0.03 for volume of left side; β = 7.18, *p* = 0.07 for volume of right side; β = 0.33, *p* = 4.86×10^-3^ for thickness of left side; β = 0.27, *p* = 0.01 for thickness of right side) and entorhinal cortex (β = 5.41, *p* = 1.44×10^-5^ for volume of left side; β = 2.08, *p* = 0.01 for volume of right side; β = 0.76, *p* = 1.55×10^-4^for thickness of left side; β = 0.80, *p* = 3.71×10^-4^for thickness of right side) (Figure 4A–4D). Only the associations of ARHL with entorhinal cortex survived the Bonferrroni adjustment. When stratified by AD continuum stage, no association was found with hippocampus volume in any group. (Figure 3A) ARHL showed significant association with higher entorhinal cortex at baseline only in the MCI and dementia stage. (Figure 4A–4D) There were no significant associations of ARHL with cortical structural measures of middle temporal region, posterior cingulate, and precuneus.

**Figure 5:**
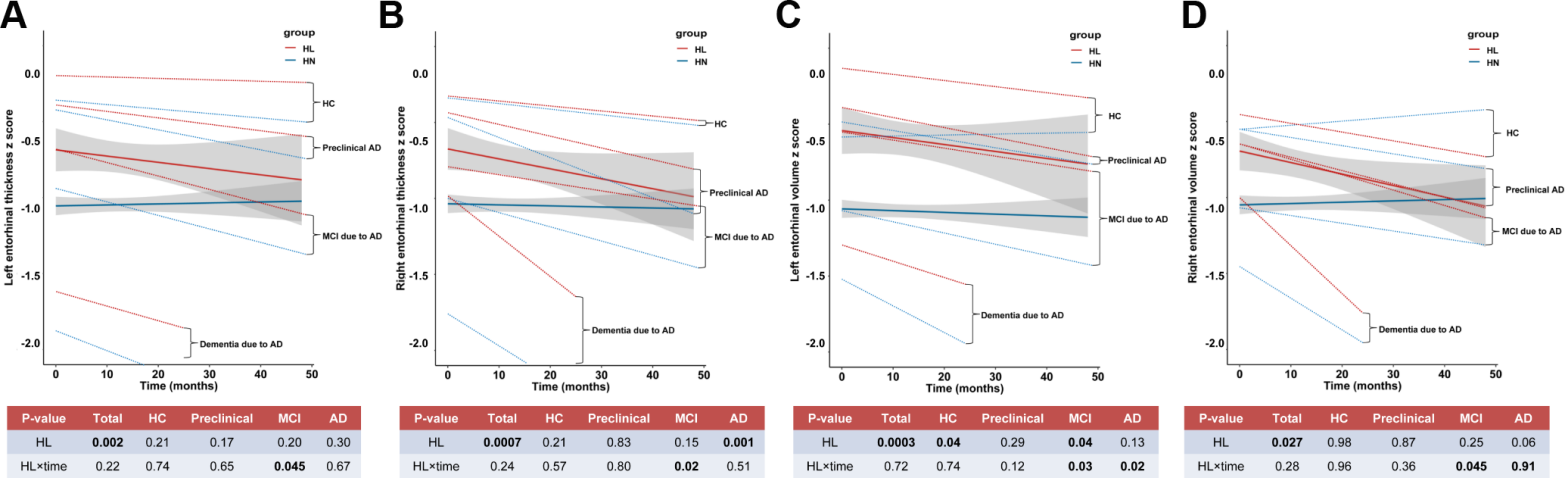
**The longitudinal influences of ARHL on structural measurements of entorhinal cortex.** ARHL at baseline was associated with more rapid cortical thinning in bilateral entorhinal cortex in prodromal AD stage (**A**–**D**). All analyses were adjusted for age, gender, education, *APOE*4 status, pathological diagnosis of AD, DM2, hypertension, hyperlipidemia, BMI, and intracranial volume. *p value was calculated for the total sample.

Moreover, the longitudinal analyses showed that ARHL at baseline was associated with more rapid cortical thinning in hippocampus (Figure 3B) and entorhinal cortex (Figure 5A–5D) in preclinical or prodromal AD, after adjusting for age, gender, education, *APOE*4 status, DM2, hypertension, hyperlipidemia, BMI, and ICV. Similar trends were observed in HC and preclinical stages.

Among those who had both cortical measures and CSF measures available (n = 822 for hippocampus; n = 491 for other ROIs), the addition of Aβ_42_, tau, and ptau_181_ in the regression of ARHL with cortical measures barely influenced the effect size (β) of ARHL. We did not identify any interaction between *APOE*4 or gender and ARHL in predicting brain imaging or CSF biomarker measures.

## DISCUSSION

Degeneration of the auditory system was reported in AD decades ago [17]. In addition to confirming the prior findings that ARHL is associated with temporal lobe atrophy [18], we demonstrated for the first time, a strong link between ARHL and the amount of tau, ptau_181_, tau/Aβ_1-42_ ratio in CSF as well as reserve capability of entorhinal cortex. These influences seemed to be more obvious in the non-demented stage of the AD continuum. We did not find a significant relationship between ARHL and Aβ levels.

Our findings are also consistent with animal histopathologic data showing that p-tau expression in hippocampus was significantly elevated after 12 months follow-up of mice with HL [19]. It can be implied that ARHL imparts influences on neurodegeneration via promoting tau pathology but not Aβ. Neuropathological and in vivo studies indicated that tau pathology could induce cognitive impairment across AD spectrum via synaptic dysfunction and neuronal loss [20], independent of amyloid pathology [21]. Notably, the negative associations might not be explained by inter-individual variations in total Aβ production because ARHL was also found not to influence CSF Aβ_1-42_/Aβ_1-40_ ratio (Supplementary Figure 4). Still, future studies should further explore the association between ARHL and amyloid in brain regions associated with the auditory function, such as the medial geniculate body (MGB) and inferior colliculus (IC), where pathological deposits were distributed throughout in AD patients [17].

Interestingly, we found subjects with ARHL had greater volume/thickness of hippocampus and entorhinal cortex at baseline. A possible explanation is that ARHL might play different roles in different stages of AD continuum. We noticed that these cross-sectional associations were only significant in clinical stage of AD continuum. On the other hand, the trends of ARHL associated with more rapid cortical thinning in hippocampus and entorhinal cortex in total group were found majoly in preclinical or prodromal stage. However, this hypothesis warrants further validation in larger studies.

There are several pathways through which ARHL may associate with increased levels of tau and ptau in CSF. Potential pathways include the SIRT1-PGC1a and LKB1-AMPK pathway that plays a role in maintaining cerebral neuron function via regulating mitochondrial function, and vascular endothelial growth factor signal pathway responsible for vascular angiogenesis and the blood–brain barrier integrity [22]. More importantly, it was shown that the expression levels of these proteins were changed in both HL and AD [22]. More animal studies are needed to shed light on the potential mechanisms via which ARHL acts in AD.

Our study had certain limitations. First, although a causal relationship between ARHL and dementia is strengthened by longitudinal analyses in the present study, the attrition bias due to loss to follow-up is not corrected in the analyses. Future studies with large sample size, longer follow-up duration, and lower attrition rate might provide more powerful evidence to support the relationship. Second, the longitudinal association can reflect, but not represent the causal relationship. Third, AHRL can contribute to depression that may exacerbate the cognitive impairment and neurodegeneration biomarker profile [23]. Although we excluded individuals with mental diseases or other neurologic conditions other than AD in the present analyses, we still cannot exclude the influences of other potential confounders, such as social isolation. Fourth, the sample size of ARHL group in some subgroups (e.g., in HC group and dementia group) was small and the results warrant further validation in larger studies. Fifth, the accurate definition of ARHL here is restricted by the information accessible from the dataset (see Supplementary Tables 1–3). As a sensitivity analysis, the results barely changed after including those who had age of onset of HL before 60.

In conclusion, our current study identified the cross-sectional as well as the longitudinal association of ARHL and the known biomarkers of AD. Our results suggested that the neurodegenerative effects of ARHL might be driven by accelerating cerebrospinal fluid tau levels and atrophy of entorhinal cortex. Furthermore, our findings suggest that prevention or management of ARHL in preclinical and prodromal stage of AD might be effective in combating neurodegeneration.

## MATERIALS AND METHODS

### ADNI

All data (including the baseline demographic characteristics, biomarkers, medical history (MH), and physical examination (PE)) were downloaded from the ADNI database (adni.loni.usc.edu). As a multicenter study, ADNI is designed to develop clinical, imaging, genetic, and biochemical biomarkers for the early detection and tracking of AD. The participants are adults aged 55–90 years with healthy control (HC), mild cognitive impairment (MCI), or mild Alzheimer’s disease (AD). Further information can be found at http://www.adni-info.org/ and in previous reports [24–29].

### Participants

**Figure 6:**
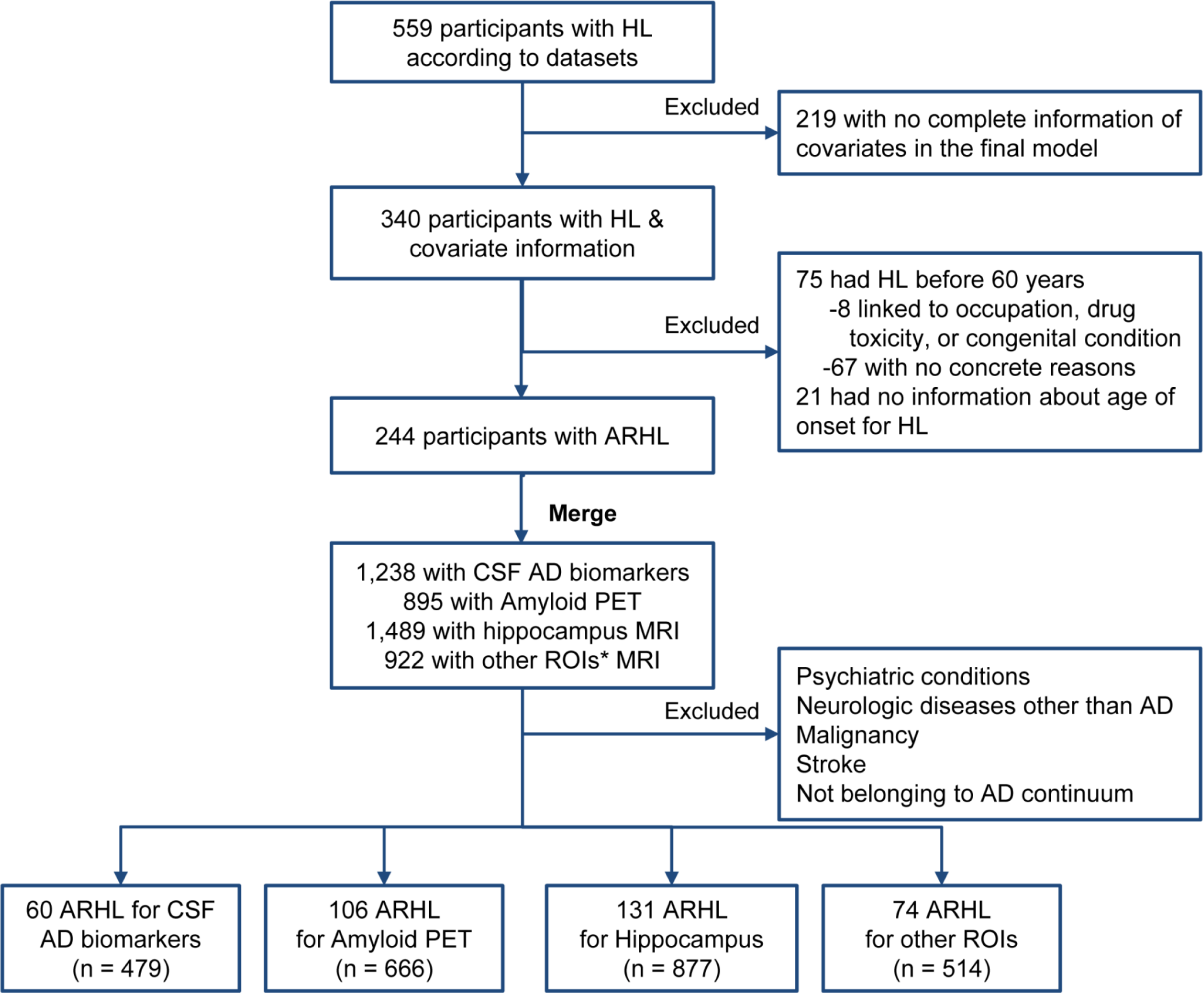
**Flow chart of searching for ARHL.** Abbreviations: HL = hearing loss; ARHL = age-related HL; CSF = cerebrospinal fluid, ROI = regions of interest. Searching terms for covariates: Hypertension: “hypert”, “HTN”, and “blood pressure”; Hyperlipemia: “lipid”, “cholesterol”, “hyperlipemia”, and “HLP”; DM2: “diabete”, “diabetic”, and “insulin”; Depression: “depress”. Other covariates (including age, gender, apoe4 status, education, diagnosis, and BMI) are accessible without searching the relevant database. ^*^Other ROIs include middle temporal cortex, entorhinal cortex, parahippocampal area, posterior cingulate, and precuneus.

The search terms used for the screening for HL include “hear”, “auditory”, “ear”, “deaf”, “presbycusis”, and “HOH (hard of hearing)”. Based on the records in MH and PE datasets, 559 participants were found to be with HL at baseline, among which 340 had information of covariates available. Furthermore, another 96 participants with HL were excluded, including 75 who had HL before 60 years and 21 who had no information about age of onset for HL, leaving 244 who had HL after 60 years old (69.7 ± 5.9, defined as age-related HL (ARHL) herein). In the subsequent analyses, we further excluded individuals with a history of psychiatric conditions (including depression), neurologic diseases other than AD, malignancy, or stroke. To restrict the study population to the AD continuum, we excluded participants with clinical diagnosis of MCI or dementia but not with evidence of cerebral Aβ deposition detected by PET (AV45 > 1.11) or CSF (Aβ < 192 pg/ml) [30]. (Figure 6) Finally, samples with data of CSF (n = 479 for Aβ42, tau, and ptau_181_), amyloid PET (n = 666), and brain structural measures (n = 877 for hippocampus; n = 514 for other regions) were included in the cross-sectional analyses. (Table 1).

### CSF measurements

CSF collection and procedural protocols have been described previously [30]. Baseline CSF Aβ_1-42_, tau, and p-tau_181_ (pg/ml) were measured using the INNOBIA AlzBio3 immunoassay (Fujirebio, Belgium). The within-batch precision values were <10% (5.1–7.8% for Aβ_1-42_, 4.4–9.8% for tau and 5.1–8.8% for ptau_181_, respectively).

### ^18^F florbetapir AV45 PET imaging

Florbetapirdata in the most fully pre-processed format was downloaded from LONI (http://adni.loni.usc.edu). The data preprocessing is accessible online (adni.loni.ucla.edu/about-data-samples/image-data/). The mean florbetapir AV45 uptake (representing the Aβ retention) within each region was calculated by co-registering the florbetapir scan to the corresponding MRI. Florbetapir SUVRs were created by averaging across four cortical grey matter regions (frontal, cingulate, lateral parietal, lateral temporal) and dividing the summary by reference region. Further details of PET acquisition and the region-of-interest protocol have been summarized previously [31].

### MRI structure

The process of MRI acquisition in ADNI has been described elsewhere [32, 33]. In brief, ADNI MRIs were acquired at multiple sites with1.5T GE, Philips, and Siemens MRI scanners using a magnetization prepared rapid acquisition gradient echo (MP-RAGE) sequence. Two high-resolution T1-weighted MRI scans were collected for each participant using a sagittal 3D MP-RAGE sequence with an approximate TR=2400ms, minimum full TE, approximate TI=1000ms, and approximate flip angle of 8 degrees. Scans were collected with a 24cm field of view and an acquisition matrix of 192 x 192 x 166 (x, y, z dimensions), to yield a standard voxel size of 1.25 x 1.25 x 1.2 mm. Images were then reconstructed to give a 256 x 256 x 166 matrix and voxel size of approximately 1 x 1 x 1.2 mm. Herein, six brain regions were defined as brain regions of interest (ROI) in the present study, including hippocampus, parahippocampal area, middle temporal cortex, entorhinal cortex, posteriorcingulate, and precuneus. The atrophy of the above regions in AD has been validated in previous MRI studies [33–38].

### Statistical analyses

Chi-square tests (for categorical variables), Student t test (for continuous variables with normal distributions), and Mann-Whitney U test (for variables with skewed distributions) were used to compare demographic, clinical, and diagnostic variables between HL and hearing normal (HN) groups. In case of skewed distribution (Shapiro-Wilk test > 0.05) of biomarker data, transformation was performed to approximate a normal distribution via “car” package of R software.

In the cross-sectional analyses, we firstly studied the associations of ARHL with CSF biomarkers (Aβ42, tau, ptau_181_, tau/Aβ42, and ptau_181_/Aβ42) after adjusting for age, gender, education, *APOE*4 status, pathological diagnosis of AD, diabetes mellitus type-2 (DM2), hypertension, hyperlipidemia, body mass index (BMI), and extracted CSF volume. Next, differences in amyloid PET and cortical structural measures (volume or thickness) between ARHL and HN were tested in 21 ROIs using a regression model adjusting for age, gender, education, *APOE*4 status, pathological diagnosis of AD, DM2, hypertension, hyperlipidemia, BMI, and intracranial volume (ICV). Significance of the HL status was examined after correction for multiple comparisons using Bonferroni method (p < 0.05). A two-way analysis of covariance model was utilized to estimate whether the association differed across the diagnostic groups. Multiple interaction terms for gender, *APOE4* status, and pathological diagnosis of AD were used to explore whether strata effect existed. In case of any potential interaction (p < 0.1), subgroup analysis was further performed. All the above analyses were repeated after stratifying by the AD continuum (preclinical AD, MCI, and dementia). Finally, to explore the influences of ARHL on the longitudinal change in the above biomarkers, we fitted linear mixed effects models to characterize individual paths of change. These models had random intercepts and slopes for time and an unstructured covariance matrix for the random effects and included the interaction between (continuous) time and ARHL as predictor. All outcome variables in linear mixed-effects models were standardized to z scores (healthy control as reference) to facilitate comparisons between modalities. Age, gender, education, *APOE*4 status, pathological diagnosis, DM2, hypertension, hyperlipidemia, BMI, extracted CSF volume or ICV were covariates.

P < 0.05 was considered significant in all analyses except where specifically noted. R version 3.5.1 and GraphPad Prism 7.00 software were used for statistical analyses and figure preparation.

### Ethics approval

The study was approved by institutional review boards of all participating institutions, and written informed consent was obtained from all participants or their guardians according to the Declaration of Helsinki (consent for research).

## Supplementary Material

Supplementary Figures

Supplementary Table 1

Supplementary Table 2

Supplementary Table 3
