# Supplementary material for: Age-related hearing loss accelerates cerebrospinal fluid tau levels and brain atrophy: a longitudinal study
Source: Aging (Albany NY). 2019 May 22;11(10):3156–69. doi: 10.18632/aging.101971 (PMC6555452; doi:10.18632/aging.101971)
Supplement: Supplementary Figures [file aging-11-101971-s001.docx]

**Supplementary Table 1. The records of ARHL definition in ADNI database (for CSF biomarker analyses, n = 60)**

| RID | Phase | Hearing aid | Ear | Onset | Description | Current status |
| --- | --- | --- | --- | --- | --- | --- |
| 188 | ADNI1 | 1 | 2 | 1996 | Bilateral hearing aid, onset 1996 | yes |
| 222 | ADNI1 | 1 | 2 | 1984 | Hearing impairment (bilateral) - corrected with hearing aids. | yes |
| 223 | ADNI1 | na | 1 | 2003 | deceased hearing in right ear | yes |
| 625 | ADNI1 | na | na | 2004 | Hearing Loss 2004 | yes |
| 644 | ADNI1 | na | na | 2003 | dec hearing 2003 | yes |
| 658 | ADNI1 | 1 | 2 | 1996 | some hearing loss-(1996)hearing aids in both ears | yes |
| 724 | ADNI1 | 1 | 2 | 1988 | Bilateral Hearing Aids, 1988 | yes |
| 725 | ADNI1 | na | 2 | 1994 | Reduced Bilateral Hearing, 1994 | yes |
| 800 | ADNI1 | na | na | 2006 | MILD HEARING LOSS-2006. | yes |
| 981 | ADNI1 | na | 2 | 2004 | bilateral decreased hearing strated 2004 | yes |
| 1097 | ADNI1 | na | na | 2001 | Mild hearing loss, onset 2001. | yes |
| 1098 | ADNI1 | na | 2 | 2006 | Hearing Loss, both ears, 01/--/2006 | yes |
| 1161 | ADNI1 | 1 | na | 2001 | b/l presbycusis. history of hearing aids for 5 years. | yes |
| 1170 | ADNI1 | na | na | 2002 | Tinnitus with mild hearing loss in 2002 | yes |
| 1292 | ADNI1 | na | na | na | moderate hearing loss due to career as Air Force pilot. Has bilateral hearing aids which work well. x35 years, approximate onset --/--/1972 | yes |
| 1373 | ADNI1 | 1 | 2 | 1994 | Bilateral Neural Hearing Loss. Has Left Hearing Aid. Onset: 1994 - Ongoing | yes |
| 2060 | ADNIGO | 1 | 2 |  | HARD OF HEARING - BILATERAL HEARING AIDS | yes |
| 2106 | ADNIGO | na | na | 2008 | Hearing loss 2008 | yes |
| 2195 | ADNIGO | na | na | na | right hearing loss, Meniere's disease; date of onset: 2002 | yes |
| 2240 | ADNIGO | na | 2 | 1997 | Bilareral hearing loss - 1997 | yes |
| 4050 | ADNI2 | na | na | na | poor hearing in right ear due to injury 40+ years ago | yes |
| 4058 | ADNI2 | na | 2 | 2009 | Hearing loss-bilateral | yes |
| 4150 | ADNI2 | 1 | na | 2007 | Hearing loss since around 2007 wears aides | yes |
| 4158 | ADNI2 | na | na | 2009 | hearing loss | yes |
| 4168 | ADNI2 | na | 2 | 1980 | Bilateral hard of hearing | yes |
| 4171 | ADNI2 | 0 | na | 2010 | has hearing aids, doesn't use them | yes |
| 4172 | ADNI2 | na | 2 | 2005 | Slight hearing loss in both ears | yes |
| 4175 | ADNI2 | 0 | 2 | 1998 | Bilateral hearing loss - refuses to use his hearing aids. | yes |
| 4176 | ADNI2 | 1 | 2 | 2001 | wears bilateral hearing aides for hearing loss | yes |
| 4177 | ADNI2 | na | na | 2006 | decreased hearing | yes |
| 4205 | ADNI2 | na | 2 | 2010 | HOH (Hard Of Hearing) Both ears | yes |
| 4250 | ADNI2 | na | 1 | 2008 | right ear hearing loss | yes |
| 4275 | ADNI2 | na | na | 2008 | Hearing loss | yes |
| 4292 | ADNI2 | na | na | na | hearing loss of high audio range in both ears from auto accident | yes |
| 4343 | ADNI2 | na | 2 | 2007 | bilateral hearing loss | yes |
| 4365 | ADNI2 | na | na | 1991 | hard of hearing | yes |
| 4386 | ADNI2 | 1 | na | 2008 | Hearing aides | yes |
| 4391 | ADNI2 | na | 2 | 2007 | mild impaired hearing Left > right | yes |
| 4392 | ADNI2 | 1 | 2 | 2006 | hearing impairment with bilateral aids | yes |
| 4396 | ADNI2 | na | 2 | 1991 | hearing loss bilateral, L>R | yes |
| 4405 | ADNI2 | na | 2 | 1996 | bilateral hearing loss | yes |
| 4444 | ADNI2 | 1 | 2 | 2006 | hearing loss, bilateral hearing aids | yes |
| 4500 | ADNI2 | na | na | 2008 | hearing loss | yes |
| 4515 | ADNI2 | na | 2 | 1980 | Hearing loss, bilaterally | yes |
| 4586 | ADNI2 | na | 1 | 2007 | Left ear hearing loss | yes |
| 4589 | ADNI2 | 1 | 2 | 1997 | bilateral hearing loss bilaterally with bilateral hearing aids | yes |
| 4595 | ADNI2 | na | 2 | 1991 | Hearing loss, b/l | yes |
| 4621 | ADNI2 | na | na | 2009 | Mild hearing loss (Date of onset: 2009) | yes |
| 4688 | ADNI2 | 1 | 2 | 2008 | Bilateral Hearing loss corrected with hearing aids | yes |
| 4815 | ADNI2 | na | na | 2006 | hearing impairment | yes |
| 4877 | ADNI2 | 1 | 2 | 2012 | hearing aids-B/L | yes |
| 4878 | ADNI2 | 1 | 1 | 2012 | Hearing loss left ear - needs hearing aid | yes |
| 4910 | ADNI2 | na | 2 | 2009 | Bilateral hearing loss | yes |
| 5112 | ADNI2 | na | na | 2011 | High frequency hearing loss | yes |
| 5141 | ADNI2 | na | na | 2011 | diminished hearing | yes |
| 5153 | ADNI2 | na | na | 1994 | Hearing Loss | yes |
| 5185 | ADNI2 | na | 2 | 2003 | Mild hearing loss - bilat | yes |
| 5237 | ADNI2 | 1 | 2 | 2003 | bilateral hearing loss with hearing aids | yes |
| 5265 | ADNI2 | 1 | 2 | 2000 | hearing loss - bilat - aids | yes |
| 5277 | ADNI2 | na | na | 2010 | mild hearing loss | yes |
