# Supplementary material for: Age-related hearing loss accelerates cerebrospinal fluid tau levels and brain atrophy: a longitudinal study
Source: Aging (Albany NY). 2019 May 22;11(10):3156–69. doi: 10.18632/aging.101971 (PMC6555452; doi:10.18632/aging.101971)
Supplement: Supplementary Table 1 [file aging-11-101971-s002.docx]

**Supplementary Table 2. The records of ARHL definition in ADNI database (for hippocampus analyses, n = 131)**

| RID | Phase | Hearing aid | Ear | Onset | Description | Current status |
| --- | --- | --- | --- | --- | --- | --- |
| 120 | ADNI1 | na | 2 | 2004 | hearing loss bilaterally-2004 | yes |
| 188 | ADNI1 | 1 | 2 | 1996 | Bilateral hearing aid, onset 1996 | yes |
| 223 | ADNI1 | na | 1 | 2003 | deceased hearing in right ear (onset 2003). | yes |
| 232 | ADNI1 | na | na | 2000 | Bilateral hearing loss (2000) | yes |
| 291 | ADNI1 | 1 | 2 | 1994 | Hearing loss bilaterally since 1994, corrected with hearing aid | yes |
| 307 | ADNI1 | 1 | 1 | 1990 | Hearing loss R side, uses aid. 1990 | yes |
| 321 | ADNI1 | 1 | na | 2004 | Hearing loss (2004), uses a hearing aid | yes |
| 481 | ADNI1 | 1 | na | 1990 | HEARING AID 1990 | yes |
| 488 | ADNI1 | 1 | 2 | 2005 | Hearing aides for bilateral hearing loss (2005), idiopathic. | yes |
| 544 | ADNI1 | 1 | 2 | 2000 | Bilateral hearing loss, onset 200, R>L, has R hearing aid. | yes |
| 610 | ADNI1 | 1 | 2 | 1991 | Bilateral hearing impairment - uses aids (onset approx. 1991). | yes |
| 625 | ADNI1 | na | na | 2004 | Hearing Loss 2004 | yes |
| 626 | ADNI1 | na | na | 1985 | Chronic Hearing Loss (--/--/1985) | yes |
| 638 | ADNI1 | na | na | 1994 | Deafness 12 years | yes |
| 644 | ADNI1 | na | na | 2003 | dec hearing 2003 | yes |
| 649 | ADNI1 | 1 | 2 | 2001 | Bilateral Hearing loss. Uses hearing aid in right ear only since 2001. Even with hearing aid, still some difficult hearing but functionally normal. | yes |
| 725 | ADNI1 | na | 2 | 1994 | Reduced Bilateral Hearing, 1994 | yes |
| 834 | ADNI1 | na | 2 | 2004 | Mild hearing loss - never diagnosed or tested formally. Onset about 2004. Left ear worse than right. | yes |
| 904 | ADNI1 | na | 2 | 1990 | Mild bilateral hearing loss diagnosed in 1990. | yes |
| 923 | ADNI1 | 1 | 2 | 2004 | Decreased Bilateral Hearing since 01/--/2004. Wears hearing aids. | yes |
| 931 | ADNI1 | 1 | 2 | 1985 | bilateral hearing loss uses hearing aid. Started 1985 | yes |
| 932 | ADNI1 | na | 1 | 2001 | decreased hearing in left ear, unknown cause, since --/--/2001 | yes |
| 1016 | ADNI1 | na | 1 | 2002 | Reduced Hearing, Left Ear, since --/--/2002. | yes |
| 1079 | ADNI1 | na | 2 | 2005 | slight hearing impairment- bilateral (2005) | yes |
| 1097 | ADNI1 | na | na | 2001 | Mild hearing loss, onset 2001. | yes |
| 1098 | ADNI1 | na | 2 | 2006 | Hearing Loss, both ears, 01/--/2006 | yes |
| 1148 | ADNI1 | na | 2 | 1998 | B/L hearing loss (1998); far sightness (1976) | yes |
| 1161 | ADNI1 | 1 | na | 2001 | b/l presbycusis. history of hearing aids for 5 years. | yes |
| 1170 | ADNI1 | na | na | 2002 | Tinnitus with mild hearing loss in 2002 | yes |
| 1203 | ADNI1 | 1 | 2 | 2004 | bilateral hearing aids (2004) | yes |
| 1373 | ADNI1 | 1 | 2 | 1994 | Bilateral Neural Hearing Loss. Has Left Hearing Aid. Onset: 1994 - Ongoing | yes |
| 1380 | ADNI1 | 1 | 2 | 1998 | Decreased bilateral hearing(1998), with bilateral hearing aids.(2002) | yes |
| 2043 | ADNIGO | 1 | na | 2003 | Hearing aids since 2003 | yes |
| 2060 | ADNIGO | 1 | 2 |  | HARD OF HEARING - BILATERAL HEARING AIDS | yes |
| 2106 | ADNIGO | na | na | 2008 | Hearing loss 2008 | yes |
| 2167 | ADNIGO | na | 2 | 1999 | Bilateral hearing loss since 1999 | yes |
| 2184 | ADNIGO | na | 2 |  | Hearing loss, bilateral | yes |
| 2210 | ADNIGO | na | 2 | 2002 | Bilateral hearing loss --/--/2002 | yes |
| 2240 | ADNIGO | na | 2 | 1997 | Bilareral hearing loss - 1997 | yes |
| 2307 | ADNIGO | na | 2 | 2010 | hearing loss bilaterally (03/2010) | yes |
| 4010 | ADNI2 | 0 | na | 2008 | Presbycusis - mild, age related hearing loss. No need for hearing aids. | yes |
| 4043 | ADNI2 | 1 | na | 2002 | Hearing loss bilateral- connected with bilateral hearing aids | yes |
| 4058 | ADNI2 | na | 2 | 2009 | Hearing loss-bilateral | yes |
| 4071 | ADNI2 | na | 2 | 2001 | Bilateral hearing loss | yes |
| 4150 | ADNI2 | 1 | na | 2007 | Hearing loss since around 2007 wears aides | yes |
| 4158 | ADNI2 | na | na | 2009 | hearing loss | yes |
| 4168 | ADNI2 | na | 2 | 1980 | Bilateral hard of hearing | yes |
| 4171 | ADNI2 | 0 | na | 2010 | has hearing aids, doesn't use them | yes |
| 4172 | ADNI2 | na | 2 | 2005 | Slight hearing loss in both ears | yes |
| 4174 | ADNI2 | na | na | 2002 | Hearing loss, minor | yes |
| 4175 | ADNI2 | 0 | 2 | 1998 | Bilateral hearing loss - refuses to use his hearing aids. | yes |
| 4177 | ADNI2 | na | na | 2006 | decreased hearing | yes |
| 4196 | ADNI2 | na | 2 | 1975 | bilateral hearing loss | yes |
| 4205 | ADNI2 | na | 2 | 2010 | HOH (Hard Of Hearing)Both ears | yes |
| 4208 | ADNI2 | na | 2 | 2005 | bilateral hearing loss | yes |
| 4218 | ADNI2 | 1 | 2 | 1998 | hearing aids for hearing loss bilaterally | yes |
| 4225 | ADNI2 | 1 | na | 2009 | wears hearing aids | yes |
| 4232 | ADNI2 | na | 1 | 2007 | Hearing loss R ear | yes |
| 4250 | ADNI2 | na | 1 | 2008 | right ear hearing loss | yes |
| 4275 | ADNI2 | na | na | 2008 | Hearing loss | yes |
| 4278 | ADNI2 | 1 | na | 1996 | HOH wears a hearing aide | yes |
| 4282 | ADNI2 | na | na | 2009 | hearing impairment | yes |
| 4291 | ADNI2 | na | 2 | 2008 | mild bilateral hearing loss | yes |
| 4294 | ADNI2 | na | 2 | 2010 | mild hearing loss bilaterally | yes |
| 4297 | ADNI2 | 1 | 2 | 2008 | Hearing Impaired- Hearing aides in both ears | yes |
| 4301 | ADNI2 | na | na | 1998 | hard of hearing | yes |
| 4302 | ADNI2 | na | na | 2007 | Hearing loss | yes |
| 4307 | ADNI2 | 1 | 2 | 2003 | bilateral diminished hearing, aided | yes |
| 4327 | ADNI2 | 1 | 2 | 1981 | bilateral hearing impairment, aided | yes |
| 4335 | ADNI2 | na | 2 | 2006 | bilateral hearing loss | yes |
| 4339 | ADNI2 | 1 | 2 | 2006 | hard of hearing (wears aides bilaterally) | yes |
| 4343 | ADNI2 | na | 2 | 2007 | bilateral hearing loss | yes |
| 4352 | ADNI2 | na | 1 | 1985 | right hearing loss | yes |
| 4357 | ADNI2 | na | na | 2004 | Hearing loss | yes |
| 4365 | ADNI2 | na | na | 1991 | hard of hearing | yes |
| 4376 | ADNI2 | na | na | 2005 | Hard of hearing | yes |
| 4386 | ADNI2 | 1 | na | 2008 | Hearing aides | yes |
| 4391 | ADNI2 | na | 2 | 2007 | mild impaired hearing Left > right | yes |
| 4392 | ADNI2 | 1 | 2 | 2006 | hearing impairment with bilateral aids | yes |
| 4396 | ADNI2 | na | 2 | 1991 | hearing loss bilateral, L>R | yes |
| 4405 | ADNI2 | na | 2 | 1996 | bilateral hearing loss | yes |
| 4410 | ADNI2 | na | na | 2008 | Hearing loss | yes |
| 4428 | ADNI2 | 1 | na | 1988 | hearing loss (wears hearing aids sporatically) | yes |
| 4438 | ADNI2 | 1 | na | 2002 | Hearing loss-hearing aids | yes |
| 4444 | ADNI2 | 1 | 2 | 2006 | hearing loss, bilateral hearing aids | yes |
| 4491 | ADNI2 | 1 | 2 | 1991 | Hearing aids, both ears | yes |
| 4500 | ADNI2 | na | na | 2008 | hearing loss | yes |
| 4515 | ADNI2 | na | 2 | 1980 | Hearing loss, bilaterally | yes |
| 4583 | ADNI2 | na | 2 | 2010 | Bilateral hearing loss | yes |
| 4586 | ADNI2 | na | 1 | 2007 | Left ear hearing loss | yes |
| 4589 | ADNI2 | 1 | 2 | 1997 | bilateral hearing loss bilaterally with bilateral hearing aids | yes |
| 4595 | ADNI2 | na | 2 | 1991 | Hearing loss, b/l | yes |
| 4621 | ADNI2 | na | na | 2009 | Mild hearing loss (Date of onset: 2009) | yes |
| 4679 | ADNI2 | na | na | 2008 | Hearing Loss | yes |
| 4688 | ADNI2 | 1 | 2 | 2008 | Bilateral Hearing loss corrected with hearing aids | yes |
| 4732 | ADNI2 | 1 | na | 2007 | HOH - uses hearing aids | yes |
| 4764 | ADNI2 | 1 | 2 | 2003 | Bilateral Hearing aids | yes |
| 4815 | ADNI2 | na | na | 2006 | hearing impairment | yes |
| 4862 | ADNI2 | na | na | 2003 | hearing impairment | yes |
| 4868 | ADNI2 | 1 | 2 | 1983 | (B) HOH; hearing aids since 1983 | yes |
| 4877 | ADNI2 | 1 | 2 | 2012 | hearing aids-B/L | yes |
| 4878 | ADNI2 | 1 | 1 | 2012 | Hearing loss left ear - needs hearing aid | yes |
| 4898 | ADNI2 | na | 1 | 2007 | Hearing loss left ear | yes |
| 4910 | ADNI2 | na | 2 | 2009 | Bilateral hearing loss | yes |
| 4936 | ADNI2 | na | na | 2003 | hearing loss | yes |
| 4938 | ADNI2 | na | na | 2003 | hearing loss | yes |
| 5014 | ADNI2 | 1 | 2 | 2002 | hard of hearing bilaterally (wears bilateral hearing aids) | yes |
| 5029 | ADNI2 | na | na | 1985 | hard of hearing (R>L) | yes |
| 5066 | ADNI2 | na | 2 | 1952 | B/L hearing loss | yes |
| 5109 | ADNI2 | na | 2 | 2005 | Bilateral hearing loss | yes |
| 5112 | ADNI2 | na | na | 2011 | High frequency hearing loss | yes |
| 5121 | ADNI2 | na | na | 2008 | high frequency hearing loss | yes |
| 5123 | ADNI2 | 1 | na | 2010 | hearing aid needed | yes |
| 5124 | ADNI2 | 1 | 2 | 2011 | Bilateral Hearing Aids- Hearing Loss | yes |
| 5141 | ADNI2 | na | na | 2011 | diminished hearing | yes |
| 5148 | ADNI2 | na | 2 | 2008 | Hearing Loss Bilaterally | yes |
| 5153 | ADNI2 | na | na | 1994 | Hearing Loss | yes |
| 5170 | ADNI2 | na | 2 | 2012 | mild hearing loss; bilateral | yes |
| 5185 | ADNI2 | na | 2 | 2003 | Mild hearing loss - bilat | yes |
| 5218 | ADNI2 | 1 | 2 | 2003 | Hearing aids - bilat | yes |
| 5236 | ADNI2 | na | 2 | 2006 | bilateral hearing loss-mild | yes |
| 5237 | ADNI2 | 1 | 2 | 2003 | bilateral hearing loss with hearing aids | yes |
| 5256 | ADNI2 | 1 | na | 2012 | hearing aids | yes |
| 5265 | ADNI2 | 1 | 2 | 2000 | hearing loss - bilat - aids | yes |
| 5271 | ADNI2 | na | 1 | 2013 | Hard of hearing, left ear | yes |
| 5275 | ADNI2 | na | na | 2005 | Sensorineural hearing loss | yes |
| 5277 | ADNI2 | na | na | 2010 | mild hearing loss | yes |
| 825 | ADNI1 | 1 | na | 2006 | HEARING AIDS - 6/--/2006 | yes |
| 828 | ADNI1 | na | 2 | 2005 | 50% hearing loss in both ears, onset 8/2005 | yes |
| 832 | ADNI1 | 1 | 1 | 1996 | hard of hearing- R. ear; wears hearing aids (1996) | yes |
