# Supplementary material for: Age-related hearing loss accelerates cerebrospinal fluid tau levels and brain atrophy: a longitudinal study
Source: Aging (Albany NY). 2019 May 22;11(10):3156–69. doi: 10.18632/aging.101971 (PMC6555452; doi:10.18632/aging.101971)
Supplement: Supplementary Table 3 [file aging-11-101971-s004.pdf]

SUPPLEMENTARY MATERIALS

Please browse Full Text version to see the data of Supplementary Tables:

Supplementary Table 1. The records of ARHL definition in ADNI database (for CSF biomarker analyses, n = 60)

Supplementary Table 2. The records of ARHL definition in ADNI database (for hippocampus analyses, n = 131)

Supplementary Table 3. The records of ARHL definition in ADNI database (for other ROIs, n = 74)

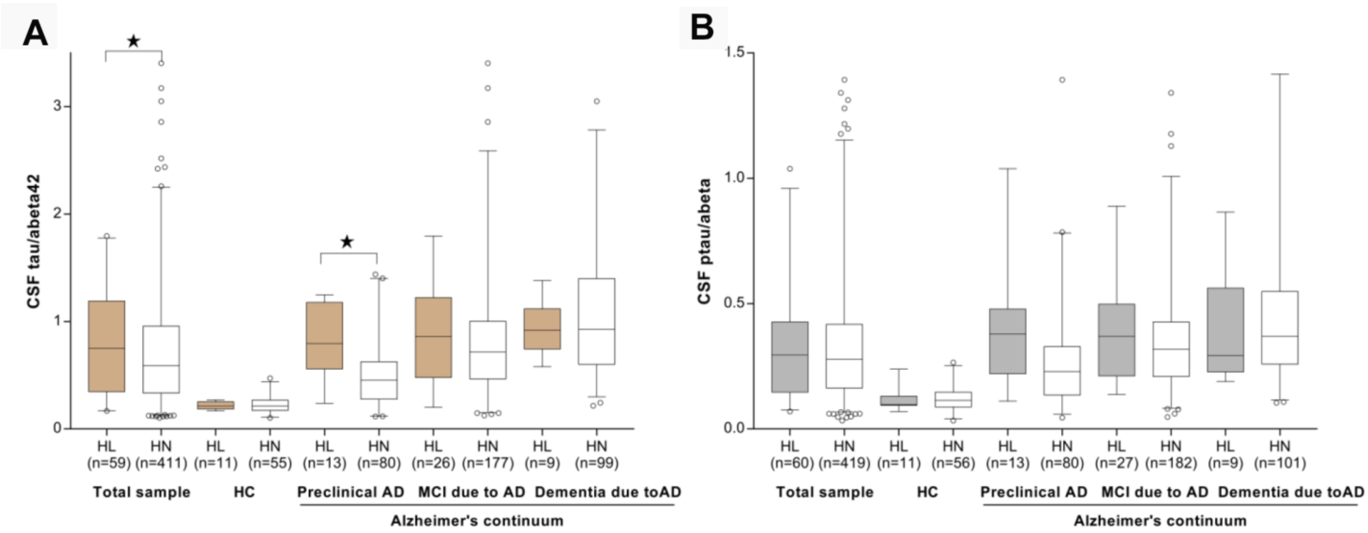

Supplementary Figure 1. The cross-sectional association of ARHL with CSF tau/Aβ_{42} and ptau$_{181}$ /Aβ_{42} ratio. ARHL was associated with higher CSF total tau/Aβ_{42} ratio (**A**), but not with ptau$_{181}$ /Aβ_{42} ratio (**B**).

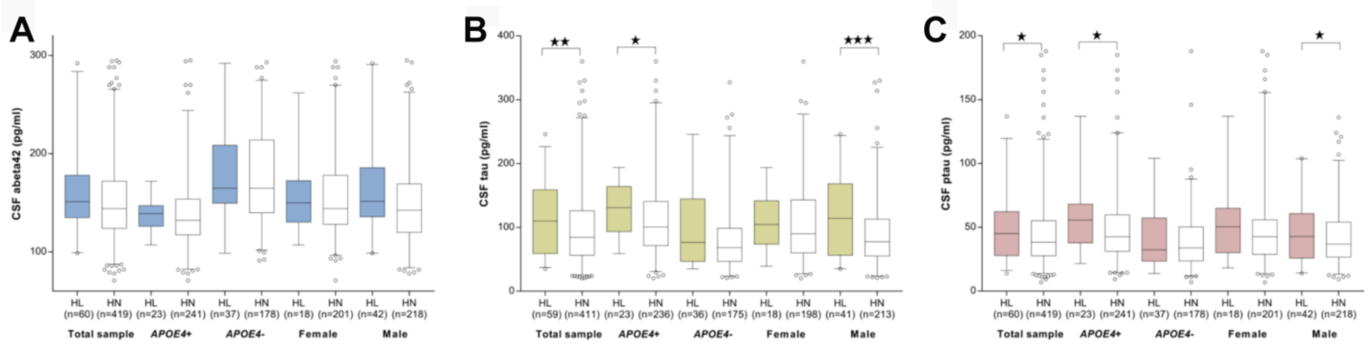

Supplementary Figure 2. Subgroup analyses according to gender and *APOE*4. Subgroup analyses showed no association of ARHL with CSF Aβ_{42} (**A**) and that ARHL was associated with greater CSF tau (**B**) or ptau$_{181}$ (**C**) only among *APOE*4 carrier (β = 0.24, p = 0.03 for tau; β = 0.25, p = 0.03 for ptau$_{181}$) and male (β = 0.30, p = 0.001 for tau; β = 0.19, p = 0.03 for ptau$_{181}$) group.

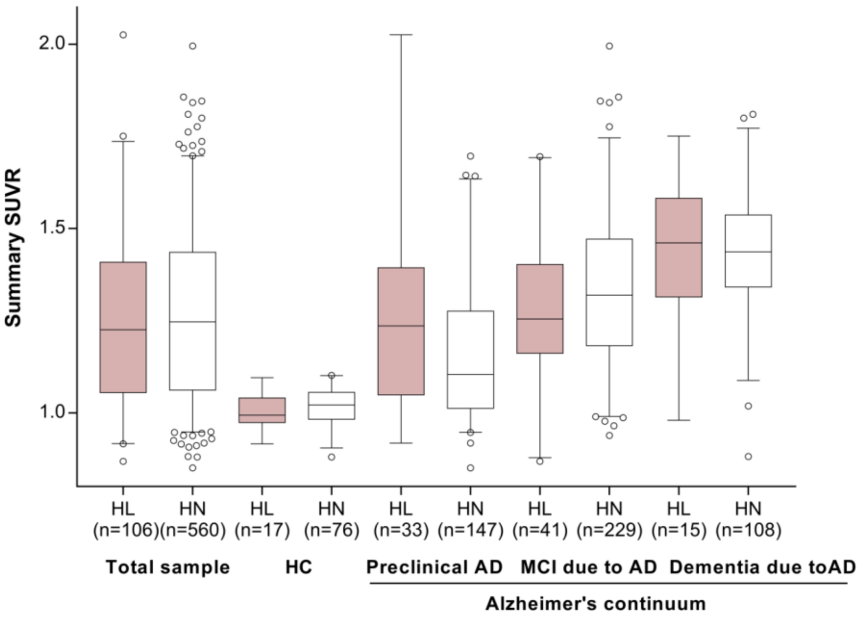

Supplementary Figure 3. The cross-sectional association of ARHL with brain amyloid deposition. No significant associations between ARHL and SUVR were found when adjusting for age, gender, education, *APOE*4 status, AD continuum group, DM2, hypertension, hyperlipidemia, and BMI.

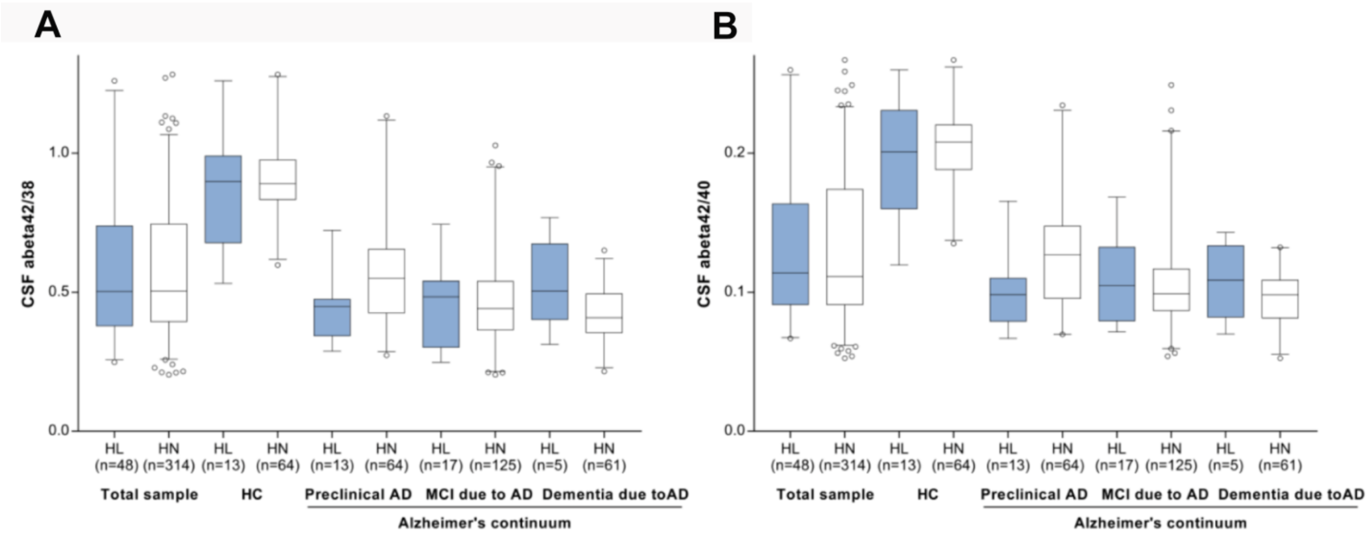

Supplementary Figure 4. The cross-sectional association of ARHL with CSF $A\beta_{42}/A\beta_{38}$ and $A\beta_{42}/A\beta_{40}$ ratio. No significant association was revealed with either $A\beta_{42}/A\beta_{38}$ (**A**) or $A\beta_{42}/A\beta_{40}$ ratio (**B**). In ADNI, CSF $A\beta_{1\text{-}42}$, $A\beta_{1\text{-}40}$, and $A\beta_{1\text{-}38}$ were measured by 2D-UPLC-tandem mass spectrometry. The overall performance of quality control is finely acceptable, with the coefficient of variation <15% (6.5-12.8%for $A\beta_{1\text{-}42}$, 2.2-7.0% for $A\beta_{1\text{-}40}$, and 5.7-8.0% for$A\beta_{1\text{-}38}$, respectively). Data was downloaded from ADNI. (HL = 48, HN = 314)
